# Model Development of a Hybrid Battery–Piezoelectric Fiber System Based on a New Control Method

**DOI:** 10.3390/polym14245428

**Published:** 2022-12-11

**Authors:** Mir Saeid Hesarian, Jafar Tavoosi, Tarek I. Alanazi

**Affiliations:** 1 Faculty of Textile Engineering, Urmia University of Technology, Urmia 5716693188, Iran; 2 Department of Electrical Engineering, Ilam University, Ilam 69315516, Iran; 3 Department of Physics, College of Science, Northern Border University, Arar 73222, Saudi Arabia

**Keywords:** piezoelectric fibers, sliding mode control, PSO, wearable energy harvesting

## Abstract

By increasing the application of smart wearables, their electrical energy supply has drawn great attention in the past decade. Sources such as the human body and its motion can produce electrical power as renewable energy using piezoelectric yarns. During the last decade, the development of the piezoelectric fibers used in smart clothes has increased for energy-harvesting applications. Therefore, the energy harvesting from piezoelectric yarns and saving process is an important subject. For this purpose, a new control system was developed based on the combination of the sliding mode and particle swarm optimization (PSO). Using this method, due to the piezoelectric yarn cyclic deformation process, electrical power is produced. This power is considered the input voltage to the controlling system modeled in this article. This system supplies constant voltage to be saved in a battery. The battery supplies power for the electrical elements of smart fabric structure for different applications, such as health care. It is shown that the presence of PSO led to the improvement of system response and error reduction by more than 30%.

## 1. Introduction

Renewable energy sources have attracted more attention for several decades. The energy obtained from mechanical energy is a kind of renewable energy. Sources such as the human body and its motion can produce electrical power as renewable energy [1,2,3,4,5]. The flexibility, low consumption of energy, and smartness of electronic devices are important subjects in studies on topics such as wearable devices for wireless short-distance communication [6,7]. The sensors and data transmitters of wearable devices consume a micro- to milli-range of watts. For example, Bluetooth transmitters need 5 mW of power to transmit a data rate of 500 kbit per second [8]. During walking, 67 W of energy can be generated and transformed from mechanical to electrical power [9]. Monitoring the vital signs of patients [10], athletes [11], and older adults [12] is the best know application of electrical stimulation. Therefore, the supply of power for wearable e-textiles and electronics [13] is an attractive topic in this field of research. Recently, low-power devices have been developed [14,15,16,17].

Heavy and bulky rechargeable batteries are used to supply power to smart textile products [18]. Because of their bulk and non-flexibility, these batteries cannot be connected to textile structures. Therefore, the development of lightweight power generation is the subject of studies [19].

Nowadays, high-tech textile products have more applications in communication, shielding, and antenna applications [20,21]. Moreover, clothes or textiles can be used as a supply source of energy [22]. Garments and fabrics with flexible, lightweight, breathable, and stretchable characteristics provide significant adaptability to deformations made by body motions. Thus, fabrics with a large surface can generate heat and mechanical energy for electrical power [23]. Therefore, developing the yarns as the basic elements in the construction of the fabric is an important subject in this field. For example, in one study, a polyester layer was curved in an elbow shape, and an output voltage of 25 V was obtained with this layer. PVDF polymer was produced by melt-spinning yarn and weaving it into fabric [24]. By compressing this fabric under 5 N, a voltage of 2.3 V was obtained. In another study, a mat constructed of multiple layers of PVDF was produced by electrospinning with different fiber alignments at different angles [25].

According to the evaluations of previous researchers in this field, it was observed that there have been few studies that have investigated harvesting and saving energy using piezoelectric yarn to be used in smart clothes. Therefore, in this article, a new control system is presented based on a voltage regulator for saving the electrical power harvested from the cyclic deformation of piezoelectric yarn. The present study was conducted for the first time. In this study, some control methods that have been implemented on hybrid systems, such as battery–photovoltaic, etc., were investigated. In [26], two PI controllers were used to adjust the output voltage of a photovoltaic–battery system. The inefficiency of the PIs in the case of wide changes in the system parameters is one of the shortcomings of this traditional controller. In [27], a battery–supercapacitor system was set up using PI–PSO hybrid control. However, the most powerful and widely used control theory for hybrid systems, including batteries, is sliding mode control [28,29]. Studies have shown that the sliding mode method has been very successful in controlling these systems and, therefore, this method was used in this study. The novelties of this study of the developed model are as follows:

1. Analysis of a combined battery–piezoelectric yarn system for the first time;
2. Tuning the SMC parameters with a new PSO-based mechanism.

Regarding the above two innovations, the first is completely new and has not been carried out before. Naturally, every study has flaws at the beginning that should be improved in the future. Regarding the second innovation, it should be noted that this item is very new and has very good performance. Adjusting the parameters of the control system with the PSO algorithm has many advantages, including increasing the accuracy and speed of the control system, not getting stuck at the local minimum points, adaptive response to changes in conditions, etc. Naturally, the manual adjustment method does not have the above advantages.

The structure of the article is such that the modeling of the combined battery–piezoelectric system is presented first. Then, the adjustable sliding mode control method with the proposed PSO is introduced. Finally, the simulation results and the conclusion are at the end of the article.

## 2. Process Modeling

**Figure 1:**
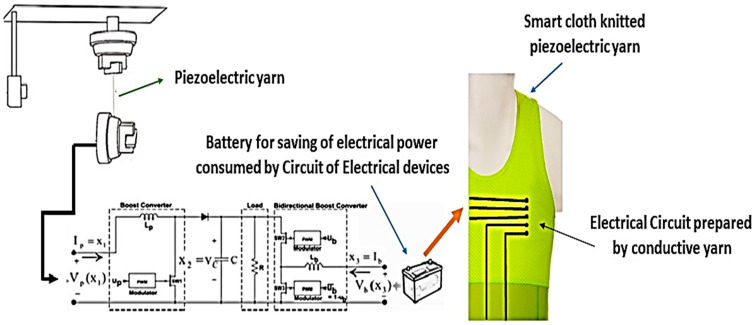
A view of the process under study.

As shown in Figure 1, the hybrid process consists of a piezoelectric yarn deformed in periodic cycles, a battery, a switching circuit, and smart fabric prepared with woven piezoelectric yarns. The function of the piezoelectric yarn is to generate mechanical energy due to the yarn deformation and transform it into electrical energy, and the function of the battery is to store this produced energy. Battery energy is used in smart clothes by electrical elements attached to the fabric structure of woven piezoelectric yarns. These smart clothes are used in different applications such as health care. The job of the converter is to compensate for the lack of power of the piezoelectric yarn. The ratio of the pulse presence of these two converters is determined by the controller.

Therefore, in this system, the controller must produce two control signals $0<u_{p}<1$ and $0<u_{b}<1$. The control signal $u_{p}$ is the presence ratio of the boost converter and is applied to the SW1 switch to adjust the current of the solar array to the desired value. The control signal $u_{b}$ is the presence ratio of the middle of the bidirectional boost and is applied to the SW2 switch to adjust the battery current to the desired value. The optimal battery current, according to the desired voltage and the principle of survival power, is calculated using Equation (1).

(1) $$P_{b}=-\left(P_{P}-P_{L}\right)\to V_{b}I_{b}=\frac{V_{C}^{2}}{R-V_{P}I_{P}}\to I_{b}=\frac{1}{V_{b}}\left(\frac{V_{C}^{2}}{R-V_{P}I_{P}}\right)$$

where $P_{b}^{\left(w\right)}$ is the output power of the battery, $P_{P}^{\left(w\right)}$ is the output power of the defamation of the piezoelectric yarns, $P_{L}^{\left(w\right)}$ is the power consumption of the load, $V_{b}^{\left(v\right)}$ is the battery voltage, $I_{b}^{\left(A\right)}$ is the current of the battery, $V_{P}^{\left(v\right)}$ is the voltage of the piezoelectric yarn, $I_{P}^{\left(A\right)}$ is the current of the piezoelectric yarn, and $V_{C}^{\left(v\right)}$ is the desired voltage of the load. The maximum power is extracted at the optimum operating point. In addition, the purpose of adjusting the battery current (according to Equation (1)) is to adjust the load voltage to the desired value. Parts (1-2), (2-2), and (2-3) of the model are the switching circuit, the piezoelectric yarn, and the battery, respectively.

### 2.1. Switching the Circuit Modeling

**Figure 2:**
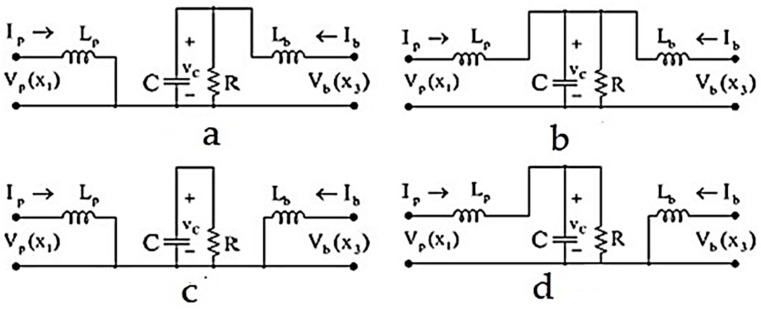
Operating modes of switching circuit. (**a**) SW1: closed, SW2: closed, and SW3: open. (**b**) SW1: open, SW2: closed, and SW3: open. (**c**) SW1: closed, SW2: open, and SW3: closed. (**d**) SW1: open, SW2: open, and SW3: closed.

The process consists of a boost converter and a bidirectional boost converter. Among the various methods of switching the circuit modeling, the two methods of moderation of state space [9] and Lagrange modeling [10] are more suitable than other methods for the controller design. In the mode method, the dynamic equations of each operating mode of the system are calculated separately, and by averaging them, the equations of the general state space of the system are obtained [9]. The switching circuit in question has four operating modes, as shown in Figure 2. The switching circuit has three switches, SW1, SW2, and SW3; the two switches SW2 and SW3 always work in full.

By calculating the state space model of each of the working modes from (a) to (d) and averaging them, the state space model is Equation (2):

(2) $$\{\begin{array}{l} {\dot{X}}_{1}=\frac{1}{L_{P}}\left(V_{P}\left(X_{1}\right)-X_{2}+X_{2}u_{P}\right)\\ {\dot{X}}_{2}=\frac{1}{C}\left(X_{1}-\frac{1}{R}X_{2}-X_{1}u_{P}+X_{3}u_{b}\right)\\ {\dot{X}}_{3}=\frac{1}{L_{b}}\left(V_{b}\left(X_{3}\right)-X_{2}u_{b}\right) \end{array}$$

where $X≜{\left[X_{1},X_{2},X_{3}\right]}^{T}={\left[I_{P},V_{C},I_{b}\right]}^{T}$ are two wins in the system mode vector. Previously, Reference [6] modeled the same process using the Lagrangian method. Comparing Equation (2) with the equations in [6], it can be seen that the results of the space mode method and the Lagrangian method are exactly the same.

### 2.2. Battery Modeling

In this paper, the Tunon method (internal resistance method) [12] was used to model the battery. In this method, the battery is modeled by Equation (3).

(3) $$V_{b}=V_{boc}-r_{b}I_{b}\;E\left(t\right)=-\int \left({\beta V}_{boc}I_{b}+W_{Loss}\right)dt,\;\beta =\{\begin{array}{cc} \beta_{1} & I_{b}>0\\ \beta_{2} & I_{b}<0 \end{array}\;SoC\left(t\right)=E\left(t\right)/E_{Max}$$

where $V_{boc}^{\left(v\right)}$ is the battery open circuit voltage, $r_{b}^{\left(Ω\right)}$ is the internal resistance of the battery, $E^{\left(J\right)}$ is the charge coefficient, $\beta_{1}$ is the discharge coefficient, $\beta_{2}$ is the energy stored in the battery, $W_{Loss}^{\left(W\right)}$ is the loss of battery storage, $E_{Max}^{\left(J\right)}$ is the maximum energy that can be stored in the battery, and SoC is the battery charge mode.

## 3. Innovative Sliding Mode Controller Design

According to Equation (4), the array operates at an optimal point, if and only if, it is shown that $s_{p}$ is the power derivative relative to the current of the piezoelectric yarn or is equal to zero in Equation (4).

(4) $$\begin{array}{cc} \frac{\partial P_{P}}{\partial I_{P}}=\frac{\partial (R_{P}I_{P}^{2})}{\partial I_{P}} & =I_{P}\left(2R_{P}+I_{P}\frac{\partial R_{P}}{\partial I_{P}}\right)=0\to S_{P}≜\left(2R_{P}+I_{P}\frac{\partial R_{P}}{\partial I_{P}}\right)\\ & =2\frac{V_{P}\left(x_{1}\right)}{x_{1}}+x_{1}\frac{\partial \left(\frac{V_{P}\left(x_{1}\right)}{x_{1}}\right)}{\partial x_{1}} \end{array}$$

where $R_{P}$ is the impedance of the piezoelectric yarn. In the SMC control method, the slip surface vector is defined according to Equation (5). The expression $S_{P}$ is optimal for placing the piezoelectric yarn at the working point and the expression $S_{b}$ is optimal for adjusting the battery current, and thus, for adjusting the load voltage (to the desired value).

(5) $$S≜\left[\begin{array}{c} S_{P}\\ S_{b} \end{array}\right]=\left[\begin{array}{c} \frac{2V_{P}\left(x_{1}\right)}{x_{1}}+x_{1}\frac{\partial \left(\frac{V_{P}\left(x_{1}\right)}{x_{1}}\right)}{\partial x_{1}}\\ x_{3}-x_{3d} \end{array}\right]$$

By solving the equation $\dot{S}=0$, the control signal will be equivalent to Equation (6) [13].

(6) $$u_{eq}=\left[\begin{array}{c} u_{Peq}\\ u_{beq} \end{array}\right]=\left[\begin{array}{c} \frac{1-V_{P}\left(x_{1}\right)}{x_{2}}\\ \frac{V_{b}}{x_{2}} \end{array}\right]$$

The SMC control signal according to Equation (7) is recommended as follows:

(7) $$u_{p}≜\{\begin{array}{cc} 0 & u_{peq}+K_{P}S_{P}\leq 0\\ u_{peq}+K_{P}S_{P} & 0<u_{peq}+K_{P}S_{P}<1\\ 1 & 1\leq u_{peq}+K_{P}S_{P} \end{array}\;u_{b}≜\{\begin{array}{cc} 0 & u_{beq}+K_{b}Sat\left(S_{b}/\phi \right)\leq 0\\ u_{beq}+K_{b}Sat\left(S_{b}/\phi \right) & 0<u_{beq}+K_{b}Sat\left(S_{b}/\phi \right)<1\\ 1 & 1\leq u_{beq}+K_{b}Sat\left(S_{b}/\phi \right) \end{array}$$

**Figure 3:**
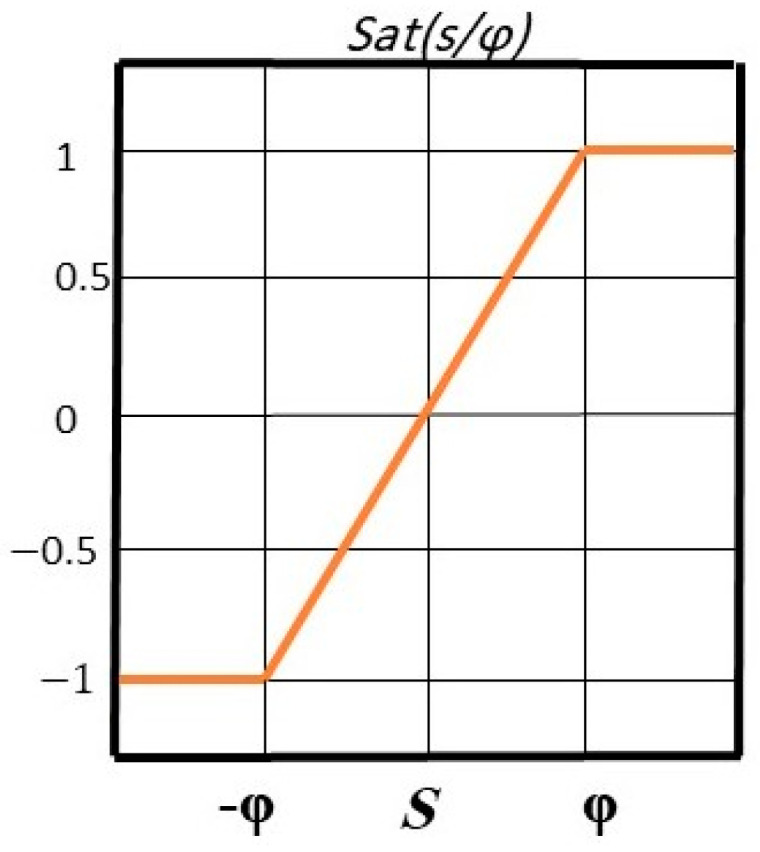
Saturation function. The horizontal axis is the angle and the vertical axis is the saturation function.

The saturation function is used to eliminate sharp fluctuations in the buzz control signal [13]. This function is shown in Figure 3.

The positive coefficients of *K_P_*, $K_{b},$ and ϕ were also selected by the PSO algorithm so that the system has good performance. According to Equation (7), it was observed that the innovative sliding mode control method did not need an optimal operating point of the piezoelectric yarn $\left(x_{1d}\right)$. As a result, the innovative controller does not need auxiliary point-tracking algorithms for maximum power.

## 4. Simulation and Testing of the Proposed Method

**Figure 4:**
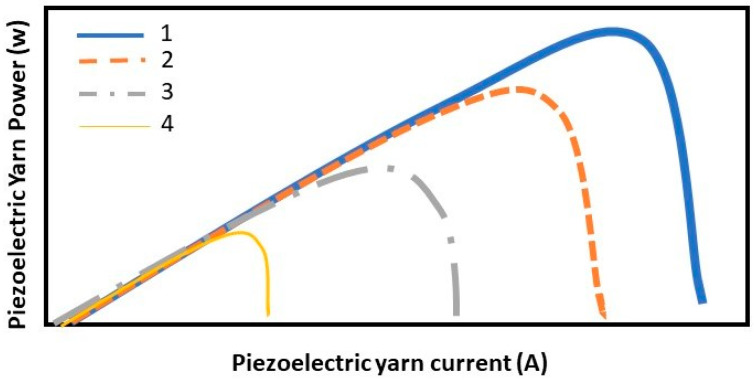
Power diagram in terms of piezoelectric yarn current under different intensities of deformations in different levels of 1>2>3>4.

To compare the trial-and-error (T&E), PSO, GA, and IGA-based sliding mode controllers, we compared their responses in solving the tuning problem. For this purpose, we defined the following criteria:

**(1) Efficiency criterion:** This criterion is defined by the cost function (8) and indicates how successful the control system has been in extracting the maximum power from the piezoelectric yarn. Obviously, the lower the cost function (8) is, the higher the efficiency of the array is. According to Figure 4, $x_{1d}$ is a current for which the power extracted from the piezoelectric yarn is maximized.

(8) $$J_{Eff}=\int_{0}^{t}{\left(x_{1}-x_{1d}\right)}^{2}dt$$

**(2) Voltage stabilization criterion:** This criterion is defined by the cost function (9) and indicates how successful the control system has been in regulating the load voltage. Obviously, the lower the cost function (9) is, the more successful the system has been in stabilizing the load voltage.

(9) $$J_{Reg}=\int_{0}^{t}{\left(x_{2}-x_{2d}\right)}^{2}dt$$

**(3) Energy storage criterion:** This criterion is defined by function (10) and indicates how successful the control system has been in energy storage. Obviously, the higher the value of the function (29), the more successful the system is in storing energy.

(10) $$\Delta SoC=SoC\left(t_{f}\right)-SoC\left(t_{0}\right)$$

where $SoC\left(t_{0}\right)$ is the initial charge status, and $SoC\left(t_{f}\right)$ is the final charge status of the battery. The simulation conditions for comparing the control methods are given in Table 1. In the simulations, each of the parameters of deformation intensity and load had step interpretations. The reference value of the load voltage was 42.5 volts. In the simulations, the required coefficients of all four control methods were obtained with a lot of trial and error in such a way that the control systems showed good performance.

**Figure 5:**
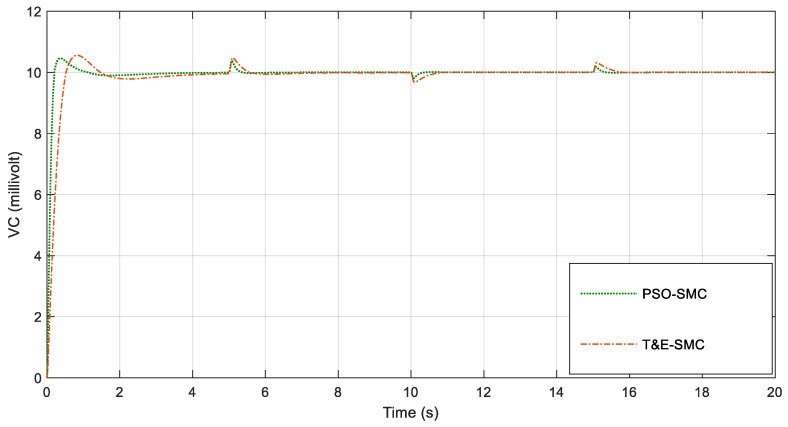
Voltage of the capacitor.

By applying the conditions of Table 1, in Figure 5, the voltage of the capacitor is shown. Note that in this figure, two sliding mode control systems based on trial-and-error and sliding mode controls adjusted with PSO are compared.

**Figure 6:**
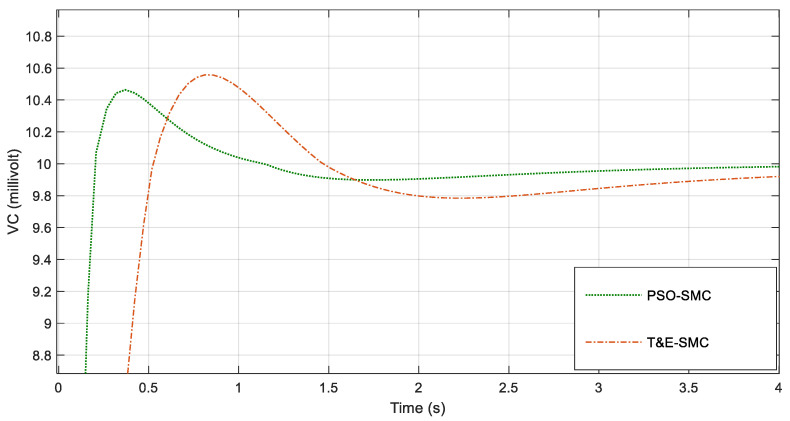
Magnification of Figure 5 around the first second.

**Figure 7:**
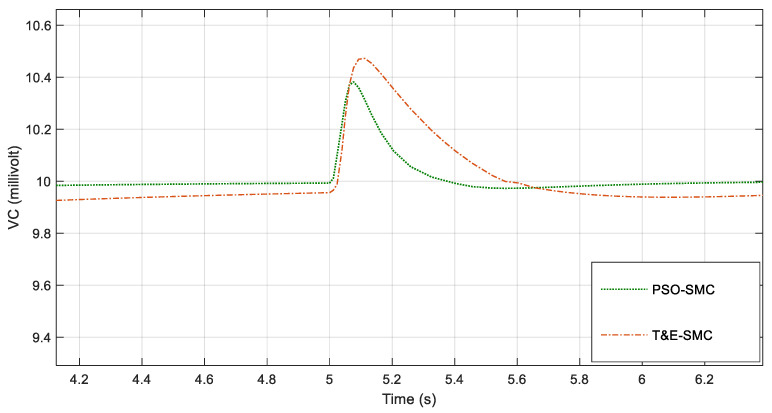
Magnification of Figure 5 around the fifth second.

**Figure 8:**
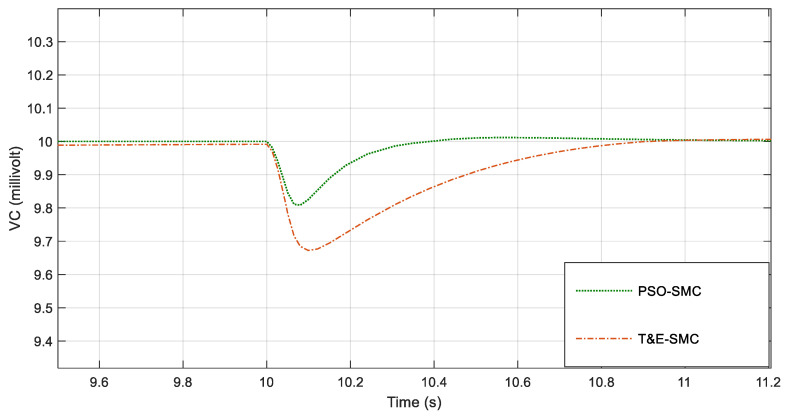
Magnification of Figure 5 around the tenth second.

**Figure 9:**
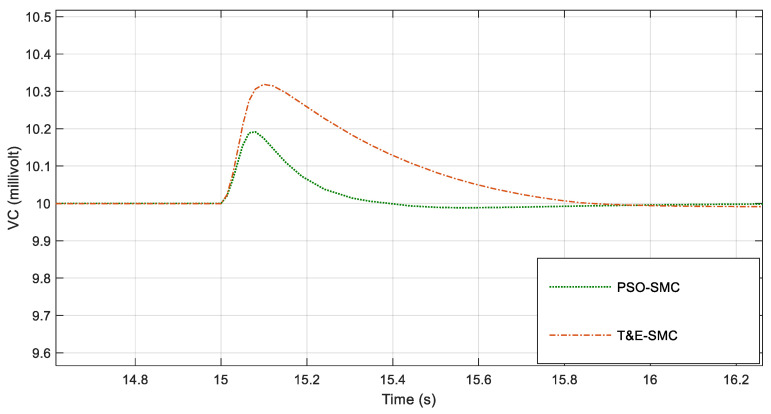
Magnification of Figure 5 around the 15th second.

For greater clarity, in Figure 6, Figure 7, Figure 8 and Figure 9, various points of Figure 5 are enlarged.

**Figure 10:**
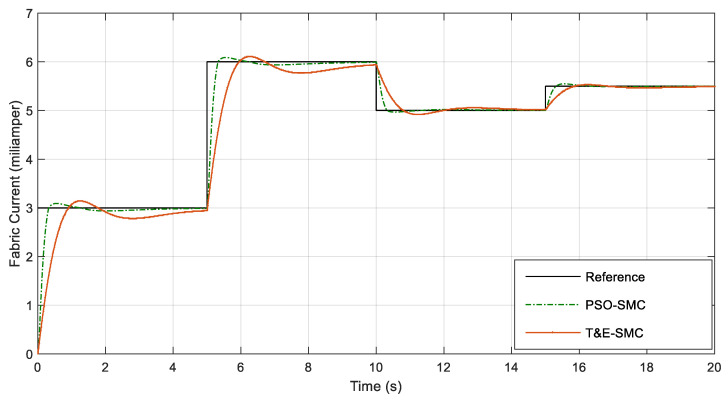
Fabric current based on the scenarios in Table 1.

As seen in Figure 6, Figure 7, Figure 8 and Figure 9, the performance of the sliding mode control system with parameters adjusted by PSO was much better and faster than the traditional sliding mode. Figure 10 shows the output current of the fabric by applying the scenarios in Table 1.

**Figure 11:**
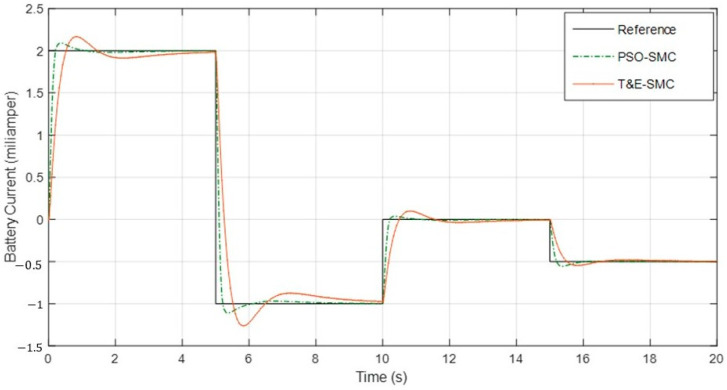
Battery current based on the scenarios in Table 1.

Figure 11 shows the output current of the battery by applying the scenarios in Table 1.

Figure 10 and Figure 11 show interesting results. As can be seen, at any moment, the sum of both currents was 5 mA and the overshoot of PSO-SMC was much less than that of trial-and-error-based SMC. Table 2 shows the numerical comparison of both control systems based on the criteria mentioned in Equations (8)–(10).

In Table 2, the effect of the presence of PSO in the sliding mode control structure is clearly evident. As can be seen, the update of the SMC parameters by PSO led to the improvement of all three evaluation criteria. Finally, in Table 3, the parameters of the battery–piezoelectric fiber system are presented as follows.

## 5. Conclusions

The energy harvested and saved using piezoelectric yarn to be used in smart clothes is a very important subject regarding the performance of this kind of garment. Therefore, in this article, a new control system is presented based on a voltage regulator for saving the electrical power harvested from the cyclic deformation of piezoelectric yarn. For this purpose, a hybrid process consisting of a battery and a piezoelectric yarn was introduced and modeled. The modeling was performed in three stages. In the first stage, the switching circuit was modeled by the mode space mode averaging method. In the second stage, the piezoelectric yarn was modeled by the single-diode method with series resistance. In the third stage, the battery was modeled by a new method. Then, an innovative controller model based on the sliding mode control designed for the global stability of the proposed controller using the appropriate Lyapunov function is introduced. To test the control methods, their performance was simulated and compared with each other. In the simulations, the two objectives of controlling the piezoelectric yarn to the optimal operating point and adjusting the load voltage to the desired value were expected. The comparison results show that the T&E control method had a long sitting time and a lot of wastage. The PSO control method also had many steady-state errors. Since environmental conditions and loads can change rapidly and in the form of bridges, the T&E control method does not have the desired efficiency and performance. The steady-state error in the PSO method can also reduce the efficiency and cause a steady-state error in the load voltage. Additionally, the nonlinear control method, namely the SMC method, showed successful performance in load opening and voltage regulation. The comparison of the cost functions showed that the PSO-based SMC control method performed better in terms of efficiency and load voltage regulation without the need to know the optimal operating point of the fabric unit.

## Figures and Tables

**Table 1:** Simulation conditions for comparison of control methods.

Time Interval (s)	Deformation Intensity (w/m^2^)	Array Temperature (°C)	Load (Ω)
0–5	500	20	100
5–10	1000	20	100
15–20	1000	40	100
20–25	1000	40	50

**Table 2:** Cost function and changes in battery charge status in different control systems.

Method	$J_{Eff}$	$J_{Reg}$	ΔSoC%
T&E *	0.0030	9.3327	+0.0782
**PSO**	0.0023	9.1764	+0.0815

* Trial and Error.

**Table 3:** The parameters of battery–piezoelectric fiber system.

Voltage (V)	Current (mA)
10	5

## Data Availability

The data presented in this study are available on request from the corresponding author.
